# Medusavirus Ancestor in a Proto-Eukaryotic Cell: Updating the Hypothesis for the Viral Origin of the Nucleus

**DOI:** 10.3389/fmicb.2020.571831

**Published:** 2020-09-03

**Authors:** Masaharu Takemura

**Affiliations:** Laboratory of Biology, Department of Liberal Arts, Faculty of Science, Tokyo University of Science, Tokyo, Japan

**Keywords:** giant virus, medusavirus, viral eukaryogenesis, origin of the nucleus, evolution

## Abstract

The mechanistic evolutionary origin of the eukaryotic cell nucleus remains unknown. Among several plausible hypotheses, the most controversial is that large DNA viruses, such as poxviruses, led to the emergence of the eukaryotic cell nucleus. Several recent findings, including the discovery of a nucleus-like structure in prokaryotic viruses and prokaryotes possessing nucleus-like inner membranes, suggest genomic DNA compartmentalization not only in eukaryotes but also in prokaryotes. The sophisticated viral machinery of mimiviruses is thought to resemble the eukaryotic nucleus: DNA replicates both inside the viral factory and nucleus, which is at least partially surrounded by membranes and is devoid of ribosomes. Furthermore, several features of the recently identified *Acanthamoeba castellanii medusavirus* suggest that the evolutionary relationship between ancestral viral factory and eukaryotic nucleus. Notably, Ran, DNA polymerase, and histones show molecular fossils of lateral transfer of nuclear genes between the virus and host. These results suggest viral innovation in the emergence of the eukaryotic nucleus. According to these results, a new scenario explaining the origin of the eukaryotic nucleus from the perspective of viral participation is proposed. This new scenario could substantially impact the study of eukaryogenesis and stimulate further discussion about viral contributions to the evolution of the eukaryotic nucleus.

## Introduction

The eukaryotic cell nucleus is a double-membrane-enclosed organelle that contains genetic material and functions as a gene expression platform. To date, although the structure, function, and various biological functions of the cell nucleus have been intensively investigated, the evolutionary origin of the cell nucleus, a milestone of eukaryotic evolution, remains unclear. Several plausible hypotheses regarding the origin of the cell nucleus have been proposed. The mainstream hypothesis is that eukaryotic cell nuclear membranes originated from cell membranes or inner membranes of proto-eukaryotic cells (Cavalier-Smith, 1987, Cavalier-Smith, 1988,Cavalier-Smith, 2010; Moreira and Lopez-Garcia, 1998; Fuerst and Sagulenko, 2012; Imachi et al., 2020), and symbiotic consequences (Martin and Koonin, 2006; Imachi et al., 2020).

However, although these hypotheses account for and explain the driving force and evolutionary pressures, they fail to portray the precise process underlying eukaryotic nucleus evolution. This process, which occurred in the period between the first eukaryotic common ancestor (FECA) and the last eukaryotic common ancestor (LECA), results in genomic DNA being surrounded by lipid bilayers; thus, compartmentalizing genomic DNA. Cavalier-Smith (1987; 1988; 2010) emphasized that the eukaryotic nucleus emerged via intracellular co-evolution, particularly concerning the cytoskeleton, heterochromatin, and nuclear pore complex. Further, Caverlier-Smith explained that the origin of the eukaryotic nucleus depends on the prior evolution of a primitive endomembrane system and primitive mitosis. The complexity of current eukaryote systems suggests that genomic DNA compartmentalization also followed the evolution of complex cellular systems, such as the inner membrane system.

The emergence of the eukaryotic nucleus is an intriguing topic that has been widely examined. The most controversial hypothesis is that large DNA viruses, such as poxviruses, led to the emergence of the eukaryotic cell nucleus (Bell, 2001; Takemura, 2001). Bell named this hypothesis “viral eukaryogenesis” (Bell, 2001). After the discovery of large DNA viruses so-called “giant virus” in 2003 (La Scola et al., 2003), an alternative theory of viral intervention in eukaryogenesis has been proposed. It is hypothesized that proto-eukaryotic cells evolved strategies alike those implemented by viruses to protect their genome against the host cell defense system, resulting in the evolution of the nucleus, which shelters the genome against viral assaults (Forterre and Prangishvili, 2009; Forterre and Gaïa, 2016). Moreover, several studies, including the synthesis of nucleus-like structures in bacterial cells by a virus, and re-construction of inner membranes of host cells by some DNA and RNA viruses, strongly supported the putative viral commitment to eukaryogenesis (Miller and Krijnse-Locker, 2008; Chaikeeratisak et al., 2017). These hypotheses, along with viral innovation theories, have received support, particularly by the discovery and characterization of the *Acanthamoeba castellanii medusavirus* (medusavirus) in 2019 (Yoshikawa et al., 2019). This review provides an update on the theory of the viral origin of the eukaryotic nucleus and discusses future research directions.

## Original Hypothesis of the Viral Origin of the Eukaryotic Nucleus

In 2001, Bell (2001) and Takemura (2001) independently proposed a new hypothesis asserting that the eukaryotic cell nucleus originated from complex DNA virus ancestors infecting ancestral eukaryotic cells. Molecular phylogenetic analysis of DNA polymerases indicated that DNA polymerases of poxviruses, the largest and most complex DNA viruses known at that time, were closely related to the eukaryotic DNA polymerase alpha, leading to this hypothesis (Takemura, 2001). In contrast to a previous study indicating that three eukaryotic replicative DNA polymerases, α, δ, and ε, evolved from ancestral ε-like DNA polymerases (Edgell et al., 1998), Takemura (2001) analyzed sequence data for B family DNA polymerases, including those of eukaryotes, bacteria, archaea, and specific DNA viruses to construct phylogenetic trees of DNA polymerases, based on the conserved regions I, II, and IV. From this analysis, Takemura inferred that eukaryotic DNA polymerase α is derived from a poxvirus-like ancestral DNA virus and further predicted that the eukaryotic nucleus had arisen via symbiotic contact of a poxvirus ancestor with an archaeote. However, this study was inconclusive because the length of amino acid sequences used for phylogenetic analysis was insufficient (Takemura, 2001).

Bell (2001) also suggested that a poxvirus is the ancestor of the eukaryotic nucleus based on several other molecular characteristics and shared features among the eukaryotic nucleus and poxviruses. Bell’s hypothesis presented eukaryotic cells as a “consortium” of three lineages replicating in concert, that is, bacteria, archaea, and viruses, and further suggesting that this consortium is the origin of meiosis and sex (Bell, 2009). The “viral eukaryogenesis” hypothesis is based partly on the similarity between poxviruses and the eukaryotic nucleus (Villarreal and DeFilippis, 2000; Bell, 2001, 2009). One similarity is mRNA capping, which is not observed in either bacteria or archaea but is a feature of new nucleo-cytoplasmic large DNA viruses (NCLDVs). Separation of the genome and ribosomes is an important feature of both poxviruses and eukaryotic nuclei (Bell, 2001). Bell considered that mRNA capping evolved in the virus to ensure the preferential translation of viral mRNA at the ribosome “outside the membrane.” This hypothesis shares analogies with that of Martin and Koonin (2006) from the perspective of the evolution of nucleocytoplasmic mechanisms, which include nuclear membrane formation and segregation of splicing and translation.

## Nucleus-Like Structures of Some Prokaryotes

One of the planctomycetes, bacterial groups belonging to the Planctomycetes, Verrucomicrobia, Chlamydiae superphylum, reportedly possess a nucleus-like cellular compartment, which contains its genomic DNA (Fuerst, 2005; Sagulenko et al., 2014). This planctomycete, *Gemmata obscuriglobus*, has a specific inner cellular structure known as the nuclear body, and a riboplasm, which are both enclosed by membranes (Sagulenko et al., 2014). Surprisingly, the inner membrane of *G. obscuriglobus* contains eukaryote-like nuclear pores (Sagulenko et al., 2017). These results suggest that *G. obscuriglobus* is a useful model of eukaryogenesis and represents evolutionary convergence (Sagulenko et al., 2017). Notable characteristics of this planctomycete are that several ribosomes are present in the nuclear body together with its genome (Fuerst and Sagulenko, 2011; Sagulenko et al., 2014), which is consistent with the absence of introns in the genome of *G. obscuriglobus*. Thus, in these membranous bacteria, it may not be necessary to exclude ribosomes from the nuclear body. These considerations suggest that the inner membranes and their complex system also existed in ancestral prokaryotic cells.

Bacteriophage 201φ2-1, which infects *Pseudomonas* sp., was found to construct a nucleus-like structure in host cells, in which bacteriophage DNA replication occurred (Chaikeeratisak et al., 2017; Hendrickson and Poole, 2018). Researchers also found that this nucleus-like structure was centered by a bipolar tubulin-based spindle like that seen in eukaryotic cell division, and newly formed viral capsids moved to the surface of the nucleus-like structure and packaged replicated DNA (Chaikeeratisak et al., 2017). In this case, the nucleus-like structure constructed by the bacteriophage 201φ2-1 was not composed of lipid bilayers but rather from several viral proteins, and the hosts were bacteria and not archaea. Thus, this is not direct evidence of the origin of the eukaryotic nucleus. However, the discovery of compartmentalization in infected cells by viruses suggests the putative presence of a similar phenomenon in the relationship between viral and eukaryotic ancestors.

## Membrane-Enclosed Viral Factory of Viruses in Host Cells

In eukaryotes, many viral interventions in the construction of a membrane-enclosed viral factory in infected cells have been reported (Miller and Krijnse-Locker, 2008). RNA viruses such as poliovirus, dengue virus, hepatitis C virus, and coronavirus construct a viral RNA replication machinery in an ER- or ER-Golgi intermediate compartment-derived own compartment. Tolonen et al. (2001) reported that replication of vaccinia virus DNA occurs at ER-enclosed cytoplasmic sites. For at least 3 h post-infection, the replication sites of the vaccinia virus in the HeLa cell cytoplasm are entirely surrounded by ER membranes, named by the authors as “mini-nuclei” (Tolonen et al., 2001; Schramm and Locker, 2005). This exciting feature of vaccinia supports both the hypothesis of the viral origin of the eukaryotic nucleus and Cavalier-Smith’s theory that the nucleus arose from the primitive endomembrane system (Bell, 2001; Takemura, 2001; Cavalier-Smith, 2010). The integration of these theories suggests that ER cisternal fusion was promoted by replication sites in ancestral DNA and RNA viruses.

The hypothesis of the viral origin of the eukaryotic nucleus has gained more attention because of the recent discovery of several NCLDVs known as “giant viruses,” which have large double-stranded DNA genomes encoding more than 400 proteins. NCLDVs possess comparatively large genome and particle sizes and have common ancestors (Iyer et al., 2006). In the International Committee on Taxonomy of Viruses (ICTV) taxonomy, NCLDVs have been proposed for re-grouping in the phylum *Nucleocytoviricota* (International Committee on Taxonomy of Viruses, 2020). Currently, 7 families belong to this group: *Phycodnaviridae*, *Ascoviridae*, *Asfarviridae*, *Iridoviridae*, *Poxviridae*, *Mimiviridae*, and *Marseilleviridae* (Iyer et al., 2006; International Committee on Taxonomy of Viruses, 2020). Among them, various types of “giant viruses,” which mainly infect to *Acanthamoeba* spp., have been isolated from various aquatic and soil environments worldwide, including the families *Mimiviridae* and *Marseilleviridae*, and genera *Pandoraviruses*, *Pithoviruses*, *Molliviruses*, *Cedratvirus*, and *Medusavirus* (La Scola et al., 2003; Raoult et al., 2004; Boyer et al., 2009; Philippe et al., 2013; Legendre et al., 2014, 2015; Abergel et al., 2015; Yoshikawa et al., 2019). The pandoravirus particle is 1 μm long and is observable by optical microscopy, as are mimiviruses. Of note, one pandoravirus has a genome size of 2.5 Mb, which is equivalent to that of the example eukaryotic, for instance, the Microsporidia genome (Philippe et al., 2013).

Several features of giant viruses suggest the involvement of a viral ancestor in the process of eukaryotic cell evolution (Takemura et al., 2015; Guglielmini et al., 2019). NCLDVs can be divided into two major superclades, named MAPI, including the families *Marseilleviridae*, *Ascoviridae*, Pitho-like viruses, and the family *Iridoviridae*, and PAM including the family *Phycodnaviridae*, *Asfarviridae*, *Mimiviridae*, and Pandoraviruses/Molliviruses (Guglielmini et al., 2019). According to the molecular phylogenetic analysis of RNA polymerase genes, it has been hypothesized that the transfer of RNA polymerase genes between proto-eukaryotic cells and the PAM superclade, resulted in the evolution of 3 RNA polymerases in eukaryotes (Guglielmini et al., 2019). Furthermore, the molecular phylogenetic analysis of DNA polymerase also suggested that gene transfer of DNA polymerase between proto-eukaryotic cells and NCLDVs (Takemura et al., 2015). These analyses suggest a major role of ancestral viruses of NCLDVs in the evolution of eukaryotic lineage.

Furthermore, the large viral factory (VF) constructed by mimiviruses in the cytoplasm of host acanthamoeba cells resembles the eukaryotic nucleus: DNA replicates both inside the VF and nucleus, which are partially or entirely surrounded by membranes. Ribosomes are excluded from the VF and nucleus, whose respective sizes are similar (Forterre and Gaïa, 2016; Hendrickson and Poole, 2018). The VF constructed by marseilleviruses is larger, but its boundary region between the VF and cytoplasm is more unclear than that of VFs constructed by mimiviruses (Boyer et al., 2009; Takemura, 2016). On the other hand, pandoravirus VF appears after the viruses-mediated degradation of the host nucleus (Philippe et al., 2013). Remarkably, mimivirus VF has been evaluated regarding its relationships between the VF and eukaryotic nucleus. Forterre et al. have discussed the similarities between the mimivirus VF and the eukaryotic nucleus and proposed several evolutionary scenarios (Forterre and Prangishvili, 2009; Forterre and Gaïa, 2016). In particular, they hypothesized that proto-eukaryotic cells recruited membrane formation mechanisms by a virus to successfully construct a nuclear envelope that shelters the cellular genome from viral attacks. In other words, our ancestral cells devised a viral-like strategy to protect its genome against the assault of viruses (Forterre and Prangishvili, 2009; Forterre and Gaïa, 2016).

Medusavirus recently found from Japanese hot spring water has unique replication strategies. Medusavirus does not construct any visible VFs in acanthamoeba cells and replicates their genome in the acanthamoeba nucleus without nuclear degradation (Yoshikawa et al., 2019). This fact led me to hypothesize that the eukaryotic cell nucleus may descend from ancestral medusavirus VFs. The idea that an ancestral VF evolved to what nowadays is known as a eukaryotic nucleus had already been suggested (Forterre and Gaïa, 2016), and has the merit of explaining the emergence of the eukaryotic nucleus. The eukaryotic nucleus degrades and is subsequently reconstructed during cell division. This dynamic performance of the eukaryotic nucleus is finely tuned by the phosphorylation and dephosphorylation of the nuclear lamina. Although how this regulatory mechanism evolved is unknown, the properties that a VF transiently constructs in host cells may be involved in the birth of the dynamic eukaryotic nucleus that is repeatedly degraded and reconstructed. This scenario is supported by the fact that viruses can modify host nuclear lamina. For example, herpes simplex viruses, which are DNA viruses phylogenetically close to NCLDV, are known to modify host lamin via its phosphorylation for their release from nuclear VF (Wu et al., 2016). This finding suggests the possibility that ancestral medusavirus, putatively one of PAM, could have had a function to modify their VF in ancestral host cells. Hence, I propose that the mechanism of transient construction of VF in ancestral PAM could be evolutionarily related to the dynamic behaviors of the eukaryotic nuclear membranes.

## Properties of *Acanthamoeba Castellanii Medusavirus*

**FIGURE 1:**
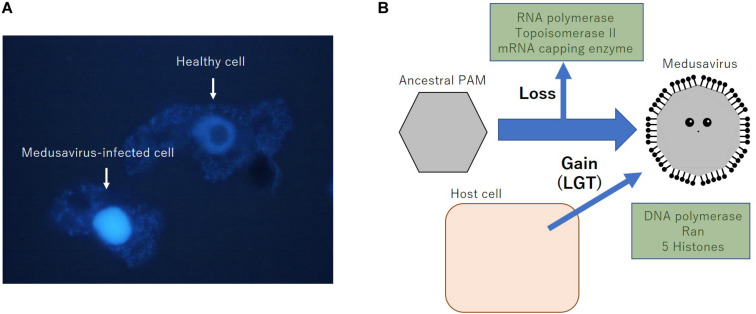
Properties of medusavirus. **(A)** Fluorescence image of DAPI-stained acanthamoeba cells with or without the infection of medusavirus. Eight hours post-infection cells were fixed by methanol and stained with 500 ng/mL of DAPI for 50 s, followed by fluorescent microscopic observation, as described previously (Takemura, 2016). **(B)** Putative scenario of loss and acquisition of several important genes of medusavirus. LGT, lateral gene transfer.

Medusavirus was discovered in hot spring water in Japan in 2019 (Yoshikawa et al., 2019). Remarkable features of medusaviruses include the absence of VFs and that they replicate their DNA in the host amoeba nucleus, indicating the co-existence of the host and medusavirus genomes in the host nucleus during viral replication. To date, several DNA viruses belonging to *Herpesviridae*, *Adenoviridae*, *Parvoviridae*, *Papillomaviridae*, and *Polyomaviridae* families are known to construct replication center (RC) in the host cell nucleus (Schmid et al., 2014). In comparison to these nuclear DNA viruses, medusavirus is known not to form any RC or VF in host nucleus, but putatively replicate their DNA using the host nuclear machinery as described below, likely in close proximity with the host genome. Fluorescence *in situ* hybridization analysis indicated that the amount of medusavirus DNA increased significantly at 8 h post-infection throughout the amoeba nucleus (Yoshikawa et al., 2019). Fluorescence of DAPI shows that the nucleus of medusavirus-infected amoeba swelled up and visible nucleolus disappeared in comparison with normal amoeba (Figure 1A). These results indicate that DNA replication of medusavirus occurs in the whole nucleus of the host cell, which is a unique characteristic among NCLDVs. However, the mechanism of packaging newly synthesized medusavirus genome in the newly synthesized empty capsid in the cytoplasm remained to be clarified. Medusavirus is the first known virus with a full set of histone-like genes in its genome, which is homologous to the full set of eukaryotic histones H1, H2A, H2B, H3, and H4 (Yoshikawa et al., 2019). The products of these genes are thought to exist in medusavirus particles, suggesting that medusavirus histone-like genes are involved in medusavirus DNA replication in the host nucleus and that both genomes co-exist because of unknown mechanisms.

It was also indicated that medusavirus had no genes for a DNA-dependent RNA polymerase, mRNA capping enzyme, or DNA topoisomerase II (Yoshikawa et al., 2019). Since all reported NCLDVs encode one or more of these enzymes in their genomes, the absence of these genes in its genome is a unique characteristic of medusavirus, which is consistent with its dependence on the host nucleus for genomic DNA replication. Furthermore, I hypothesize that medusavirus has lost these genes during the evolution of a nucleus-dependent DNA replication mechanism while acquiring a full set of histone genes from the host cell (Figure 1B).

The phylogenetic tree placed medusavirus B family DNA polymerase at the root of a clade of eukaryotic DNA polymerase δ, suggesting that the medusavirus B family DNA polymerase gene was more closely related to the eukaryotic DNA polymerase δ than to other giant virus family B DNA polymerase genes, suggesting a close evolutionary relationship between medusavirus and its host, *A. castellanii* (Yoshikawa et al., 2019). This relationship indicates that lateral gene transfers between the virus and host occurred and that the ancestral medusavirus acquired DNA polymerase δ from its host, along with a full set of histones. Further, the phylogenetic analysis of the major capsid protein (MCP) gene of medusavirus showed that the medusavirus MCP gene formed a monophyletic group with MCP genes in the amoeba genome, suggesting that lateral gene transfer of MCP genes had anciently occurred from medusavirus to amoeba (Yoshikawa et al., 2019). These lateral gene transfers are thought to occur more easily when a viral genome co-exists with a host genome as in the case of medusavirus than when a viral genome is separated from the host genome.

**FIGURE 2:**
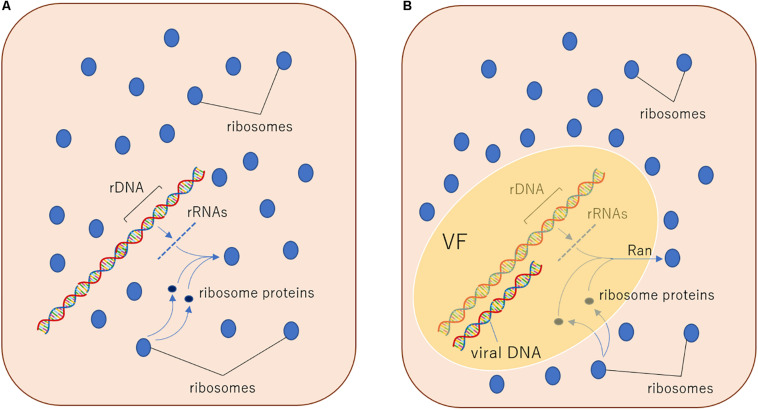
Hypothetical evolutionary mechanisms of the exclusion system of ribosomes from the nucleus. **(A)** In a putative ancestral prokaryotic host cell, DNA genome and ribosomes co-exist in proximity, so transcribed rRNA genes can directly construct ribosome particles near DNA because of the absence of intron and splicing system. **(B)** In a putative ancestral PAM (here hypothesize medusavirus)-infected host cell, DNA genome was separated from ribosomes by the function of putative Ran outside VF, because of the presence of intron and splicing system. This putative VF may have evolved into the eukaryotic nucleus. VF, viral factory.

The eukaryotic GTPase Ran plays a role in the ribosomal translocation from the nucleus to the cytoplasm. Ribosomal proteins with a nuclear export signal are recognized by exportins and exported to the cytoplasm dependent on the GTPase activity of Ran (Moy and Silver, 1999). Interestingly, medusavirus possesses a homologous gene of this eukaryotic Ran in its genome (Yoshikawa et al., 2019). Members of the family *Mimiviridae* also have Ras-related protein genes; however, in medusavirus, this gene is more closely related to eukaryotic Ran compared to other virus Ras-related protein genes (Yoshikawa et al., 2019). In the molecular phylogenetic tree, the position of medusavirus Ran is within the clade of the eukaryotic Ran (Yoshikawa et al., 2019), suggesting that medusavirus was acquired Ran gene from its eukaryotic host. According to this result, I hypothesize that the export mechanism of ribosomal proteins through the nuclear membrane emerged when a proto-nuclear envelope was developed from VF of ancestral PAM, followed by the acquisition of the Ran gene by ancestral medusavirus (Figure 2). Additionally, many giant viruses, including medusavirus and the family *Mimiviridae*, contain introns in several genes. Medusavirus has 7 genes, including introns (Yoshikawa et al., 2019). Although the function of medusavirus Ran remains unknown, the ancestral medusavirus also includes an intron, and therefore export of ribosomal subunits from the inner to the outer VF is an essential process for the separation of splicing and translation (Figure 2). Based on this information, the ancestral Ran protein, which is involved in excluding ribosomes from the VF, may have also evolved into the current Ran protein.

## A New Hypothesis of Eukaryogenesis

The evolution of the eukaryotic nucleus was likely a complicated process. To explain this event, several important processes should be included and described based on evidence. Besides the origin of the “function” of the eukaryotic nucleus, such as DNA replication, transcription, splicing, and ribosomal exclusion, the origin of the “materials” for the structure of the eukaryotic nucleus, such as nuclear lamins, lamin-associated proteins, nuclear membranes, and nuclear pore complex, can be partly explained. These structures originated through symbiotic contact of ancestral mitochondria with the ancestral eukaryotes according to several molecular phylogenetic analyses. Mans et al. (2004) suggested that several central components of the nuclear pore complex have a bacterial endosymbiotic origin, including Ran, the HEH domain of Src1p-Man1, key domains of karyopherins, and nucleoporins. Furthermore, the immunoglobulin (Ig) domains in animal lamins and the nuclear membrane protein GP210 have been associated with distinct prokaryotic families of Ig domains (Mans et al., 2004). The proto-eukaryotic nucleus containing a proto-nuclear pore complex and proto-nuclear lamina may have emerged from an ancestral VF of PAM in combination with these endosymbiont-derived factors.

**FIGURE 3:**
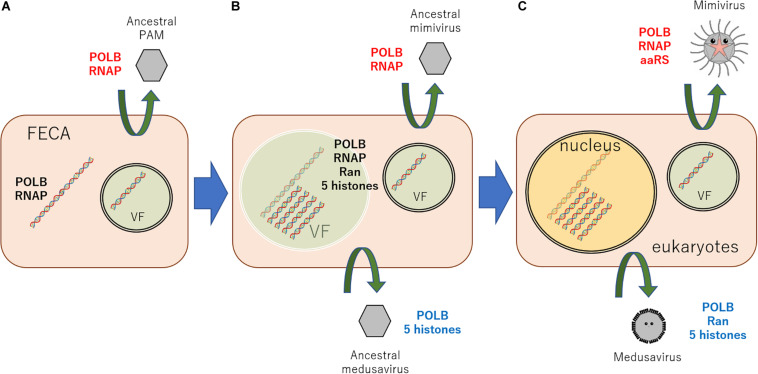
New scenario explaining the origin of the eukaryotic nucleus. **(A)** An ancestral virus of the currently existing giant viruses, PAMs, constructed a VF, which surrounded the viral genome using a cytoplasmic inner membrane (putatively ER)-derived membranes in infected proto-eukaryotic cells. **(B)** Among these ancestral viruses, viruses replicating its DNA very closely to host genome emerged, putatively ancestral medusavirus. Against this viral “attack,” the host cell developed a defense system to envelope its genome with the inner membrane. **(C)** This membrane evolved into what nowadays is known as the nuclear membrane. After the emergence of the nucleus, giant viruses infecting eukaryotic cells evolved and diversified to several currently present infection systems. Black genes represent host cell genes, red genes indicate mimivirus lineage genes, and blue genes stand for medusavirus lineage genes. POLB, B family DNA polymerase; RNAP, DNA-dependent RNA polymerase; FECA, the first eukaryotic common ancestor; VF, viral factory.

According to these results, a new scenario can be hypothesized, explaining the origin of the eukaryotic nucleus (Figure 3). An ancestral virus of currently existing giant viruses of PAM superclade constructed a VF in the cytoplasm of proto-eukaryotic cells, which surrounded by a cytoplasmic inner membrane (putatively ER)-derived membranes. Meanwhile, the host cell has developed membrane-derived compartmentalization to protect genomic DNA from viral attacks. This compartment was, however, transiently constructed in response to viral infection. This primordial mechanism led the evolutionary birth of several genes, nowadays known as histones and Ran, which proved useful for DNA condensation, and molecular shuttling between the inner and outer compartments. I speculate that a portion of viral DNA replication may have aided host genome replication and proved evolutionary advantageous. Ancestral medusavirus, which replicated its DNA physically close to the host genome, have evolved from ancestral PAM. This event stimulated the formation of a “semi-permanent” nucleus in host cells to shelter genomic DNA from viral nucleases and also the exclusion of matured ribosome outside membrane using ancestral Ran genes. It is hypothesized that several lateral gene transfers between host DNA and ancestral medusavirus DNA, which replicated its DNA in semi-permanently-constructed host cell “nucleus,” resulted in the acquisition of eukaryotic δ-like DNA polymerase, Ran, and a full set of histone genes by medusavirus genome. On the other hand, ancestral mimivirus, which replicated its DNA in ER-derived cytosolic VF, and other PAM members have evolved independently. Additionally, several proteins, such as bacteria-originated ancestral lamins described above, might have participated in the evolution of the eukaryotic nucleus structure. These evolutionary events, which links FECA to LECA, along with PAM evolution, and several lateral gene transfers, including DNA and RNA polymerases between cells and viruses, must have occurred, resulting in the diversification of NCLDVs (Takemura et al., 2015; Guglielmini et al., 2019). After the emergence of the nucleus, ancestral PAM infecting eukaryotic cells evolved and diversified to current viruses with several infection systems, such as the current medusavirus, pandoraviruses, and mollivirus, as well as *Phycodnaviridae*, *Mimiviridae*, and *Asfarviridae* families.

Although the properties of mimivirus VFs resemble those of the eukaryotic nucleus, an important point has not been solved: how have VFs surrounded not only the viral genome but also the host genome? Medusavirus may provide insight into this unsolved problem. Medusavirus contains several genes essential for the function of the eukaryotic nucleus. Phylogenetic analysis suggests that these genes were acquired from the host via lateral gene transfer. Medusavirus DNA replication occurs in the host cell nucleus, suggesting a relationship between the current eukaryotic nucleus and a speculative ancestral medusa VF, and that a medusa VF may surround both the host and viral genomes. However, this is only a hypothetical scenario based on the properties of medusavirus, which is presently considered as the most appropriate example of a viral factor putatively involved in the viral origin of the eukaryotic nucleus. Some unsolved questions regarding the evolutionary linking between viruses and the emergence of the eukaryotic nucleus remain open. For instance, how several types of introns, including self-splicing introns, have emerged and how that can be interpreted from the viewpoint of viral commitment? However, this new scenario can profoundly impact the study of eukaryogenesis and provide a basis for further discussion on the involvement of viruses in the evolution of the eukaryotic nucleus. Nevertheless, additional studies on the mechanism of medusavirus infection and the life cycle in detail should be conducted in the future. Furthermore, more appropriate and attractive viruses or giant viruses, which may explain the commitment of viruses in the emergence of the eukaryotic nucleus, should be identified and investigated.

## Data Availability Statement

The original contributions presented in the study are included in the article, further inquiries can be directed to the corresponding author.

## Author Contributions

The author confirms being the sole contributor of this work and has approved it for publication.

## Conflict of Interest

The author declares that the research was conducted in the absence of any commercial or financial relationships that could be construed as a potential conflict of interest.
